# The Relationship Between Depression, Anxiety, and Stress Levels and Eating Behavior in Emergency Service Workers

**DOI:** 10.7759/cureus.35504

**Published:** 2023-02-26

**Authors:** Selime Celik Erden, Banu Karakus Yilmaz, Nalan Kozaci, Abdullah Burak Uygur, Yavuz Yigit, Kadir Karakus, Ismail Erkan Aydin, Tugce Ersahin, Durmus Ali Ersahin

**Affiliations:** 1 Department of Psychiatry, Alanya Alaaddin Keykubat University, Faculty of Medicine, Antalya, TUR; 2 Department of Emergency Medicine, Alanya Alaaddin Keykubat University, Faculty of Medicine, Antalya, TUR; 3 Department of Emergency Medicine, Hamad Medical Corporation, Doha, QAT

**Keywords:** depression-anxiety-stress level, sociodemographic factors, restricted eating, external eating, emotional eating, emergency service workers

## Abstract

Introduction

It is seen that shift work causes various biological, psychological, and behavioral problems in individuals. This study aimed to determine the eating attitudes and behaviors of health workers working in shifts in a stressful environment such as the emergency service and to examine the relationship between depression, anxiety, and stress levels and eating behaviors (emotional eating, restrictive eating, and external eating) in terms of sociodemographic and clinical characteristics.

Material and Methods

Sociodemographic data form; Depression, Anxiety, and Stress Scale (DASS); and Dutch Eating Behavior Questionnaire (DEBQ) were used. The study sample consisted of 92 employees (doctor, nurse, emergency medical technician (EMT), medical secretary, and security, staff) who were actively on duty in the emergency department of Alanya Alaaddin Keykubat University Medical Faculty Training and Research Hospital.

Results

In our study, when the eating behavior of emergency service workers was evaluated in terms of "emotional, external, and restricted eating" sub-dimensions, depression (p=0.043), anxiety (p=0.017), increased stress levels (p=0.002), being female (p=0.022), nurse-emergency medical technician profession (p=0.001), working in 24-hour shifts (p=0.001), and diet history (p=0.013) were associated with "emotional eating." In addition, an increase in depression levels (p=0.048), being single (p=0.015), working in 24-hour shifts (p=0.005), a decrease in age (p<0.001) with "extrinsic eating," an increase in body mass index (BMI) (p=0.020) and waist circumference (p=0.049), and diet history (p<0.001) were associated with "restricted eating."

Conclusions

In our study, among the sociodemographic factors, being female, being single, working in 24-hour shifts, diet history, nurse-EMT profession, and undergraduate education level were found to increase the tendency to develop eating behavior problems. An increase in depression levels, being single, working in 24-hour shifts, and a decrease in age were associated with "extrinsic eating." There is a correlation between depression, anxiety, and stress scores and emotional eating scores. Additionally, we found significant correlations between body mass index, waist circumference, diet history, and restricted eating scores. In the approach to eating behavior problems, it is important to determine the individual eating behavior disorder. Due to the increased risk of eating behavior disorder in those who work in long shifts such as 24 hours, it will be possible to organize work programs and increase the quality of service.

## Introduction

An important part of the factors that disrupt the physiological and psychological balance of individuals, causing stress, originates from work life [1]. Working in the emergency department requires being in contact with many patients with different conditions and their relatives and making the right clinical decisions, in a timely manner. Emergency health service employees work in shifts to provide 24-hour uninterrupted health service. Shift work can adversely affect the work and personal life of individuals by disrupting the health of individuals at various levels physiologically and psychologically [2]. It is reported that the shift work system causes both stress and a decrease in the skill levels of coping with stress [3]. Stressful work environments cause a wide variety of psychiatric problems. One of the most important problems that may occur due to shift work is changes in eating behavior and eating disorders [4].

Numerous studies investigating the relationship between stress and eating behavior have shown that stress is associated with changes in food intake in adults and children [4]. While the increase in glucocorticoids caused by acute stress may decrease hypothalamic-pituitary axis activity and eating, in the case of chronic stress, high glucocorticoids can increase food intake by acting as a stimulant.

It has been reported that high levels of stress are associated with a number of binge-eating episodes and excessive weight gain [5]. It has been reported that stress affects the amount of food consumed and the selected food types [4], causing an increase in the consumption of unhealthy foods, especially those with high fat and sugar content [6].

Individuals use different coping methods to cope with the stressor. Emotional eating behavior can act as a coping mechanism for many. It is thought that those with emotional eating behavior are more likely to overeat under stress than those without emotional eating behavior [7]. In addition, it has been reported that individuals with emotional eating behavior increase, in response to stress factors, food cravings and the consumption of foods containing high carbohydrates and fats [7]. Depressive symptoms and anxiety symptoms, such as stress, are psychiatric conditions that are highly associated with changes in eating behavior such as the loss of appetite and overeating. A number of studies, using different instruments to measure emotional eating, have also demonstrated an association between binge eating and weight gain in response to negative emotions (depressed and anxious) [8]. Studies have shown that individuals with depressive symptoms generally have dysfunctional coping strategies and are prone to develop abnormal eating behaviors accompanied by episodes of binge eating to reduce negative mood states [8].

Disordered eating behavior refers to problematic eating behaviors such as binge eating or purging and restrictive eating that are less frequent and less severe than those required for a diagnosis of an eating disorder and may be a leading marker for an eating disorder diagnosis [9]. Impaired eating behaviors, other than eating disorders such as anorexia nervosa, bulimia, and binge eating, which can be diagnosed with direct signs and symptoms, can be ignored as a health problem. It is seen that shift work causes various biological, psychological, and behavioral problems in individuals. This study aimed to determine the eating attitudes and behaviors of health workers working in shifts in a stressful environment such as the emergency room and to examine the relationship between depression, anxiety, and stress levels and eating behaviors (emotional eating, restrictive eating, and external eating) in terms of sociodemographic and clinical characteristics.

## Materials and methods

Study group

Between November 2022 and December 2022, 98 employees (doctor, nurse, emergency medical technician (EMT), medical secretary, security staff, and nursing aide) were included in the study. Employers who were at least primary school graduates did not use psychotropic drugs for at least one month for any reason and gave written consent to participate in the study. They were actively on duty in the emergency department of Alanya Alaaddin Keykubat University Medical School.

After giving informed consent, participants filled out the sociodemographic questionnaire; Depression, Anxiety, and Stress Scale (DASS); and Dutch Eating Behavior Questionnaire (DEBQ) [3]. Those who had difficulties in performing the tests applied in the study and had cognitive impairment to a degree that made it difficult to comply with the research guidelines, those who did not actively work in the emergency room during the study period, and those with alcohol and substance use disorders were excluded from the study. Six participants were excluded from the study (four due to antidepressant use and two for incomplete filling of the forms), and study was performed with 92 participants. All participants were informed about the research by the two expert psychiatrists who conducted the study, their written consent was obtained, and they were evaluated according to DSM-5 criteria, and Structured Clinical Interview for DSM-5 (SCID-5-CV) was administered [10], because of the exclusion of another psychiatric disease.

Scales used in the research

Sociodemographic Questionnaire

It is a form created by researchers who question the demographic characteristics of the participants (age, gender, educational status, and marital status) and their characteristics such as body mass index (BMI), waist circumference, and diet history.

DASS

DASS is a self-report scale developed by Lovibond and Lovibond (1995) to assess participants' symptoms of depression, anxiety, and stress [11]. It consists of three subgroups (depression, anxiety, and stress) and contains a total of 21 items. The Turkish validity and reliability study of the scale was conducted by Sarıçam (2018) [12]. The Cronbach alpha coefficients of the subgroups of DASS-21, which have three factors, depression, anxiety, and stress, were determined as 0.84 for anxiety, 0.87 for depression, and 0.85 for stress. High scores obtained in the application of the scale indicate that the severity of the symptoms increases.

DEBQ

DEBQ is developed by Van Strien et al. [13], and the Turkish validity and reliability study of the questionnaire was conducted by Bozan et al. (2011) [14]. A questionnaire of 33 items, it consists of three subscales that assess emotional eating behaviors (e.g., do you eat sweets when you are unhappy?), external eating behaviors (e.g., do you eat more than you normally would if your food smells very good?), and restricted eating behaviors (e.g., do you eat less than you would like to not gain weight?). The items in the questionnaire are evaluated with a five-point Likert scale. Although the scale does not have a cutoff point, the high total scores reflect a negativity related to the eating behavior [13,14].

Ethics committee approval

Ethics committee approval of the study was obtained from the Non-Interventional Clinical Research Ethics Committee of the Alanya Alaaddin Keykubat University Medical Faculty Training and Research Hospital, with the decision number of 2022/10-03. An informed consent form was signed by all participants, and the study was conducted in accordance with the Declaration of Helsinki.

Statistical analysis

Descriptive Statistics

Mean and standard deviation were used for normally distributed continuous variables and frequencies and percentages for interquartile range and categorical variables. To determine the normality of continuous variables, Shapiro-Wilk and Kolmogorov-Smirnov tests were used. For normally distributed independent variables, the difference between groups for DEBQ scores was compared with Student's t-test. For the comparison of DEBQ scores for professions, the one-way analysis of variance (ANOVA) test was performed, and the pairwise comparisons were performed with the post hoc Tukey-Tukey's B honestly significant difference (HSD) test. Tests for the correlation of DEBQ scores with DEBQ scores and other parameters were calculated by the partial correlation analysis and controlled for confounding variables. To evaluate all analyses, a 95% confidence interval and a significance level of p<0.05 were determined. The statistical analyses were made using the Statistical Package for Social Sciences (SPSS) 22.0 (IBM SPSS Statistics, Armonk, NY) software.

## Results

The sociodemographic characteristics and clinical scale scores of the participants

Of the 92 participants (mean age of 31.65±7.97), 42.4% were female, and 57.6% were male. Their average working time in the emergency department was 5.19±5.51 years, and their average monthly working time was 13.88±4.68 days. While the average body mass index of the participants was 25.64±4.70 kg/m^2^, the average waist circumference was 34.28±5.36 inches (Table 1).

**Table 1:** The sociodemographic characteristics and clinical scale scores of the participants n, number of participants; SD, standard deviation; DASS, Depression, Anxiety, and Stress Scale; DEBQ, Dutch Eating Behavior Questionnaire

		n	%
Profession	Medical Secretary	10	10.9
	Emergency Medical Technician	5	5.4
	Nurse	27	29.3
	Physician	26	28.3
	Security Staff	6	6.5
	Nursing Aide	13	14.1
	Others	5	5.4
Sex	Female	39	42.4
	Male	53	57.6
Education	Primary School	2	2.2
	High School	15	16.3
	University	22	23.9
	Bachelor's Degree	53	57.6
Marital Status	Single	59	64.1
	Married	32	34.8
	Divorced/Widow	1	1.1
Shift Type (Hour)	24	61	66.3
	8-16	7	7.6
	12-24	22	23.9
	16-8	2	2.2
Waist Circumference (cm)		87.07	13.61
Diet History	No	47	51.1
	Yes	45	48.9
Alcohol Consumption	No	48	52.2
	Yes	44	47.8
Smoking	No	37	40.2
	Yes	55	59.8
		Mean	SD
Age		31.65	7.97
Body Mass Index (kg/m^2^)		25.64	4.70
Working Duration in the Emergency Room (Year)		5.19	5.51
Average Working Duration per Month (Day)		13.88	4.68
DASS Scale: Depression Score		6.55	4.99
DASS Scale: Anxiety Score		4.04	3.84
DASS Scale: Stress Score		6.60	4.93
DASS Scale: Total Score		17.20	12.54
DEBQ: Restricted Eating Score		23.90	8.23
DEBQ: Emotional Eating Score		27.27	13.67
DEBQ: External Eating Score		31.71	6.93
DEBQ: Total Score		82.47	20.85

Comparison of Dutch Eating Behavior Questionnaire scores based on sociodemographic characteristics

The DEBQ emotional eating scores (6.917, p=0.022) and total scores (11.251, p=0.010) were higher in females compared to males. The DEBQ total scores were (9.268, p=0.034) higher in those with a bachelor's degree compared to those without. Comparing married participants, single participants had a higher external eating (4.006, p=0.015) total score (9.246, p=0.042). Emotional eating (9.070, p=0.001), external eating (4.228, p=0.005), and total DEBQ scores (12.770, p=0.005) were higher in those who work 24-hour shifts, comparing to others. The DEBQ restricted eating scores (7.760, p<0.001), emotional eating scores (7.080, p=0.013), and total eating scores (17.659, p<0.001) were higher in those with a diet history, compared to those without (Table 2).

**Table 2:** Comparison of Dutch Eating Behavior Questionnaire (DEBQ) scores based on sociodemographic characteristics SD, standard deviation; EMT, emergency medical technician

DEBQ Scores and Sociodemographic Characteristics		Mean	SD	Mean Difference	95% Confidence Interval		χ^2 ^Value/Fisher	p
					Lower Limit	Upper Limit		
Restricted Eating Score	Female	25.00	9.24	1.906	-1.675	5.486	4.288	0.292
	Male	23.09	7.39					
Emotional Eating Score	Female	31.26	15.59	6.917	1.041	12.793	5.154	0.022
	Male	24.34	11.34					
External Eating Score	Female	32.69	6.63	1.711	-1.188	4.610	0.578	0.244
	Male	30.98	7.11					
DEBQ Total Score	Female	88.95	20.99	11.251	2.784	19.717	0.398	0.010
	Male	77.70	19.60					
Restricted Eating Score	Not University Graduate	22.46	9.18	-2.501	-5.933	0.932	1.643	0.151
	University Graduate	24.96	7.37					
Emotional Eating Score	Not University Graduate	24.95	14.88	-4.032	-9.731	1.666	1.206	0.163
	University Graduate	28.98	12.56					
External Eating Score	Not University Graduate	30.13	7.24	-2.740	-5.604	0.124	1.040	0.061
	University Graduate	32.87	6.51					
DEBQ Total Score	Not University Graduate	77.13	21.54	-9.268	-17.840	-0.696	0.417	0.034
	University Graduate	86.40	19.61					
Restricted Eating Score	Single	24.50	8.21	1.719	-1.866	5.303	0.115	0.343
	Married	22.78	8.29					
Emotional Eating Score	Single	28.43	12.50	3.340	-2.597	9.276	1.746	0.267
	Married	25.09	15.60					
External Eating Score	Single	33.10	6.02	4.006	0.824	7.189	4.115	0.015
	Married	29.09	7.81					
DEBQ Total Score	Single	85.68	18.96	9.246	0.335	18.157	1.966	0.042
	Married	76.44	23.11					
Restricted Eating Score	Others	23.29	9.14		20.10	26.48	0.146	0.865
	Nurse-EMT	24.31	8.51		21.24	27.38		
	Physician	24.19	6.79		21.45	26.94		
Emotional Eating Score	Others	21.24	9.10		18.06	24.41	6.919	0.002
	Nurse-EMT	32.94	16.28		27.07	38.81		
	Physician	28.19	12.26		23.24	33.14		
External Eating Score	Others	29.35	6.84		26.97	31.74	3.287	0.042
	Nurse-EMT	33.25	6.04		31.07	35.43		
	Physician	32.88	7.46		29.87	35.90		
DEBQ Total Score	Others	73.38	17.47		67.29	79.48	6.398	0.003
	Nurse-EMT	90.50	22.54		82.37	98.63		
	Physician	84.46	18.73		76.90	92.03		
Restricted Eating Score	24-hour shift	23.67	7.60	-0.683	-4.310	2.945	0.586	0.709
	Others	24.35	9.48					
Emotional Eating Score	24-hour shift	30.33	14.45	9.070	4.032	14.107	4.381	0.001
	Others	21.26	9.63					
External Eating Score	24-hour shift	33.13	6.93	4.228	1.305	7.151	0.249	0.005
	Others	28.90	6.10					
DEBQ Total Score	24-hour shift	86.77	21.05	12.770	3.980	21.561	0.971	0.005
	Others	74.00	17.90					
Restricted Eating Score	No Diet History	20.11	7.29	-7.760	-10.784	-4.737	0.001	<0.001
	Diet History	27.87	7.29					
Emotional Eating Score	No Diet History	23.81	11.09	-7.080	-12.626	-1.535	5.541	0.013
	Diet History	30.89	15.22					
External Eating Score	No Diet History	30.72	6.45	-2.010	-4.867	0.847	0.991	0.166
	Diet History	32.73	7.32					
DEBQ Total Score	No Diet History	73.83	16.83	-17.659	-25.521	-9.797	2.783	<0.001
	Diet History	91.49	20.98					
Restricted Eating Score	No Alcohol	22.69	8.73	-2.540	-5.933	0.854	0.708	0.141
	Alcohol	25.23	7.53					
Emotional Eating Score	No Alcohol	26.27	15.03	-2.093	-7.775	3.590	2.338	0.466
	Alcohol	28.36	12.08					
External Eating Score	No Alcohol	31.13	6.88	-1.216	-4.094	1.663	0.072	0.404
	Alcohol	32.34	7.00					
DEBQ Total Score	No Alcohol	79.29	21.91	-6.640	-15.223	1.942	0.700	0.128
	Alcohol	85.93	19.28					
Restricted Eating Score	Nonsmoker	23.41	9.11	-0.831	-4.326	2.664	3.580	0.638
	Smoker	24.24	7.66					
Emotional Eating Score	Nonsmoker	28.95	15.22	2.800	-2.976	8.577	2.800	0.338
	Smoker	26.15	12.53					
External Eating Score	Nonsmoker	32.92	6.88	2.028	-0.885	4.941	0.031	0.170
	Smoker	30.89	6.90					
DEBQ Total Score	Nonsmoker	84.81	23.89	3.920	-5.389	13.229	4.065	0.403
	Smoker	80.89	18.60					

Comparison of Dutch Eating Behavior Questionnaire scores based on professions

The DEBQ emotional eating score (p=0.002), external eating score (p=0.042), and total scores (p=0.003) of the participants who were divided into three groups as nurse-emergency medical technician, doctor, and other emergency service workers (security, staff, and secretary) were significantly different. In order to determine which groups have difference, the statistical significance level was reduced to p≤0.017, and the pairwise comparisons were performed with the post hoc Tukey-Tukey's B HSD test (Table 3). Emotional eating scores (11.702, p=0.001) and DEBQ total scores (17.118, p=0.002) were higher in nurse-emergency medical technician group compared to other emergency service personnel.

**Table 3:** Comparison of Dutch Eating Behavior Questionnaire (DEBQ) scores based on professions SE, standard error; CI, confidence interval; EMT, emergency medical technician

Clinical Scale Scores	Professions		Mean Difference	SE	95% CI		p
					Lower Limit	Upper Limit	
DEBQ Restricted Eating Score	Others	Nurse-EMT	-1.018	2.048	-6.76	4.72	0.873
		Doctor	-0.898	2.167	-6.97	5.17	0.910
	Nurse-EMT	Others	1.018	2.048	-4.72	6.76	0.873
		Physician	0.120	2.196	-6.03	6.27	0.998
	Physician	Others	2.167	2.167	-5.17	6.97	0.910
		Nurse-EMT	2.196	2.196	-6.27	6.03	0.998
DEBQ Emotional Eating Score	Others	Nurse-EMT	-11.702	3.167	-20.58	-2.83	0.001
		Physician	-6.957	3.350	-16.34	2.43	0.101
	Nurse-EMT	Others	11.702	3.167	2.83	20.58	0.001
		Physician	4.745	3.395	-4.77	14.26	0.346
	Physician	Others	6.957	3.350	-2.43	16.34	0.101
		Nurse-EMT	-4.745	3.395	-14.26	4.77	0.346
DEBQ External Eating Score	Others	Nurse-EMT	-3.897	1.666	-8.56	0.77	0.056
		Physician	-3.532	1.762	-8.47	1.40	0.117
	Nurse-EMT	Others	3.897	1.666	-0.77	8.56	0.056
		Physician	0.365	1.786	-4.64	5.37	0.977
	Physician	Others	3.532	1.762	-1.40	8.47	0.117
		Nurse-EMT	-0.365	1.786	-5.37	4.64	0.977
DEBQ Total Score	Others	Nurse-EMT	-17.118	4.856	-30.72	-3.51	0.002
		Physician	-11.079	5.137	-25.47	3.31	0.084
	Nurse-EMT	Others	17.118	4.856	3.51	30.72	0.002
		Physician	6.038	5.206	-8.55	20.62	0.480
	Physician	Others	11.079	5.137	-3.31	25.47	0.084
		Nurse-EMT	-6.038	5.206	-20.62	8.55	0.480

Correlation of Depression, Anxiety, and Stress Scale scores with Dutch Eating Behavior Questionnaire scores

DASS depression scores were positively correlated with DEBQ emotional eating scores (r=0.194 and p=0.070) and external eating scores (r=0.219 and p=0.041). DASS anxiety scores were positively correlated with DEBQ emotional eating scores (r=0.245 and p=0.021) and DEBQ total scores (r=0.245 and p=0.021). DAS stress scores show a positive correlation with DEBQ emotional eating scores at the level of r=0.316, positively (p=0.002), and with DEBQ total scores at a level of r=0.319 (p=0.002). DASS total scores were positively correlated with DEBQ emotional eating scores (r=0.277 and p=0.009) and DEBQ total scores by (r=0.272 and p=0.010) (Table 4).

**Table 4:** Correlation of Depression, Anxiety, and Stress Scale (DASS) scores with Dutch Eating Behavior Questionnaire (DEBQ) scores

		DASS Depression Score	DASS Anxiety Score	DASS Stress Score	DASS Total Score	Restricted Eating Score	Emotional Eating Score	External Eating Score
DASS Depression Score	r	1						
	p							
DASS Anxiety Score	r	0.693	1					
	p	0.000						
DASS Stress Score	r	0.791	0.712	1				
	p	0.000	0.000					
DASS Total Score	r	0.922	0.866	0.929	1			
	p	0.000	0.000	0.000				
DEBQ Restricted Eating Score	r	-0.008	0.162	0.126	0.097	1		
	p	0.945	0.131	0.242	0.368			
DEBQ Emotional Eating Score	r	0.194	0.245	0.316	0.277	0.290	1	
	p	0.070	0.021	0.003	0.009	0.006		
DEBQ External Eating Score	r	0.219	0.090	0.175	0.183	-0.014	0.335	1
	p	0.041	0.405	0.103	0.087	0.895	0.001	
DEBQ Total Score	r	0.176	0.245	0.319	0.272	0.587	0.880	0.531
	p	0.100	0.021	0.002	0.010	0.000	0.000	0.000

Correlation of anthropometric and work characteristics with Dutch Eating Behavior Questionnaire scores

Age shows a negative correlation with DEBQ external eating scores (-0.428, p=0.000). Body mass index shows a positive correlation with DEBQ restricted eating scores (r=0.284 and p=0.008). Waist circumference was positively correlated with DEBQ restricted eating scores (r=0.271 and p=0.011) (Table 5).

**Table 5:** Correlation of sociodemographic characteristics with Dutch Eating Behavior Questionnaire (DEBQ) scores

		DEBQ Restricted Eating Score	DEBQ Emotional Eating Score	DEBQ External Eating Score	Age	Working Duration in the Emergency Room (Year)	Average Working Duration per Month (Day)	Body Mass Index (BMI)	Waist Circumference
DEBQ Restricted Eating Score	r	1							
	p								
DEBQ Emotional Eating Score	r	0.201	1						
	p	0.061							
DEBQ External Eating Score	r	-0.053	0.370	1					
	p	0.628	0.000						
Age	r	0.106	-0.156	-0.428	1				
	p	0.329	0.150	0.000					
Working Duration in the Emergency Room (Year)	r	-0.089	-0.013	-0.229	0.651	1			
	p	0.411	0.906	0.033	0.000				
Average Working Duration per Month (Day)	r	-0.019	-0.235	-0.151	0.159	0.031	1		
	p	0.859	0.028	0.163	0.141	0.775			
Body Mass Index (BMI)	r	0.284	0.050	-0.113	0.114	0.183	0.020	1	
	p	0.008	0.644	0.299	0.293	0.090	0.856		
Waist Circumference	r	0.271	-0.082	-0.196	0.209	0.194	0.051	0.879	1
	p	0.011	0.448	0.069	0.052	0.072	0.639	0.000	
DEBQ Total Score	r	0.513	0.871	0.575	-0.202	-0.103	-0.216	0.098	-0.022
	p	0.000	0.000	0.000	0.061	0.344	0.045	0.364	0.839

## Discussion

In our study, we found that females, singles, those with a bachelor's degree, nurses and emergency medical technician group, those who worked in 24-hour shifts, and those with a history of diet had higher DEBQ total scores. On the other hand, restricted eating scores were high for only the ones with a history of diet, which are single participants and those who worked in 24-hour shifts. In addition, while there was a significant positive relationship between restricted eating scores and body mass index and waist circumference, a significant negative relationship was found between age and extrinsic eating scores.

When the relationship between depression, anxiety, and stress levels and eating behavior is evaluated, a positive and significant relationship was found between DASS total scores and DEBQ total scores. Similar to our results, it has been shown in many studies that emotional eating is more common in females than in males [15]. Also, there are studies showing that being slim in females is idealized due to factors such as social environment and media, and this can facilitate the development of eating behavior disorders; also, emotion-focused coping styles that females use more can cause eating disorders such as impaired eating behavior and binge-eating disorder [16]. Our results show that female employees working in a stressful environment such as the emergency room have a greater risk of developing eating behavior disorders compared to male colleagues.

In our study, external eating scores and total eating scores were higher in single participants compared to married ones. In the study by Bussolotti et al., who reported that being married is a negative feature for eating disorders, it was reported that interpersonal functionality should be considered rather than being married or single in the development of eating disorders [17]. In our study, higher eating scores were determined in participants with undergraduate education compared to participants with undergraduate education level. Participants who had a bachelor's degree had higher eating scores compared to participants with a lower degree of education. However, we did not find any difference when comparing the subgroups (emotional, external, and restricted eating) with each other.

In the study by Ulusoy (2022) conducted in Turkey, it was reported that the emotional eating scores of individuals with university and higher graduate degrees were higher than the participants with lower degrees of education [18]. In the study by Gökensel-Okta et al. (2022), higher restricted eating scores were reported in university graduates than in high school and secondary school graduates [19]. Sample selection can be the reason for the different results of these studies. Also, a limitation of our study is the fact that most of the participants in our study had a bachelor's degree may have affected our results and the generalizability of the study.

In our study, emotional eating, external eating, and total eating scores were found to be higher in emergency service workers who work in "24-hour" shifts. Studies have reported that shift workers tend to eat more meals and snacks later in the day and consume more calories and fat, including sweets, sugary drinks, and low-fiber foods, in their diets [20].

In our study, emotional eating and total eating scores were higher in nurses and emergency medical technicians compared to other participants. Similar to our study results, a cross-sectional study of Canadian nurses working in shifts reported an increase in the frequency of snack consumption and eating behavior problems [21]. The work environment can be an important consideration when assessing the impact of diet on health outcomes in a shift worker population. It has been reported that nurses change their diet regimens, especially after they start working in shifts [22]. In our study also, we found that especially, nurses have a more tendency to develop eating disorders among emergency service workers.

Studies done in different countries investigating the relationship between age and eating behavior show that restricted eating behavior [23] and emotional eating behavior decrease with age [24]. In our study, we found that external eating decreases with age. In a study conducted in Spain, consistent with our study results, the only eating behavior associated with age was external eating, which decrease with age [25]. Our results suggest that individuals tend to prefer healthy foods rather than external characteristics such as appearance and smell in their nutritional preferences as they get older.

In the absence of alternative behaviors, eating can be considered as a natural reward or satisfaction habit in order to cope with negative emotions. In our study, we show that there is a correlation between depression, anxiety, and stress scores and emotional eating scores. Emotional eating has been associated with depressive symptoms, particularly atypical depression [26]. Unlike depressive disorders accompanied by the loss of appetite, depression with atypical features is characterized by increased appetite, which can lead to weight gain [10]. Individuals with atypical depression tend to develop abnormal eating behaviors such as emotional eating, that is, overeating in response to negative emotions.

In their study, Ozier et al. reported that emotional eating may be a mediating factor between depression and body mass index [26]. Contrary to these studies, no relationship was found between body mass index and emotional eating in our study. In our study, the mean BMI of the participants was close to normal (25.64±4.70), and our results are in line with the study by Geliebter and Aversa, who reported that individuals with a BMI below 25 kg/m^2^ reduced their food intake in negative emotional states while individuals with a BMI above 25 kg/m^2^ increased their food intake [27].

In our study, a significant correlation was found between body mass index, waist circumference, diet history, and restricted eating scores. Obesity, which is the most basic findings of an increase in body mass index and waist circumference, is a health problem often accompanied by depression and anxiety, as well as psychological eating patterns such as emotional eating, addictive eating behaviors, and binge eating. Studies in the literature mostly focus on the relationship between emotional eating and obesity. The results of our study indicate that restricted eating may also be associated with obesity.

Studies have reported that dietary restriction may be the most important predictor of overeating during stress [28]. In our study, emotional eating, restricted eating, and total eating scores were found to be higher in emergency service workers with a diet history, which is consistent with the literature. In situations where changes in eating habits are observed under stressful conditions, behavioral patterns have been shown to occur in two opposite ways. In situations where stress is experienced chronically, a person may increase food intake in response to stress, which can lead to weight gain or decreased food intake, which can lead to weight loss [29]. There is no clear consensus in the literature as to whether stress leads to increased or decreased calorie intake, but it seems likely that stress is associated with changes in food choice, such as higher-calorie desserts and fatty foods [30]. In their study investigating the relationship between stress and eating behavior, Snoek et al. found strong evidence showing a decrease in the consumption of vegetables at main meals and an increase in the consumption of high-calorie snack foods between meals in individuals with higher stress levels [30]. In our study, stress scores were correlated with emotional eating and total eating scores, in line with the literature. Well-being trainings to be given to individuals working in the emergency department to cope with stress will contribute significantly to the reduction of the tendency to develop eating behavior problems and eating disorders in this specific group.

Strength and limitation

Our study has a small sample size and was conducted in one center only, which complicates the generalizability of our results. Future studies with larger sample sizes including multiple centers are necessary in this field. To the best of our knowledge, our study is the first article to evaluate primary eating behaviors and related factors in emergency service workers in our country. The fact that our study examined eating behavior problems in a specific sample group, such as emergency service workers, rather than the general population constitutes one of the unique aspects of our study. Another strength of our study is that emergency workers who are actively on duty were included in the study, and all participants were evaluated by two psychiatrists.

## Conclusions

In our study, among the sociodemographic factors, being female, being single, working in 24-hour shifts, diet history, nurse-EMT profession, and undergraduate education level were found to increase the tendency to develop eating behavior problems. At the same time, when evaluated in terms of "emotional, external, and restricted eating" sub-dimensions, being a female, nurse-EMT profession, working in 24-hour shifts, and diet history show a relationship with "emotional eating." An increase in depression levels, being single, working in 24-hour shifts, and a decrease in age were associated with "extrinsic eating." There is a correlation between depression, anxiety, and stress scores and emotional eating scores. Additionally, we found significant correlations between body mass index, waist circumference, diet history, and restricted eating scores. Restricted eating may also be associated with obesity.

In the approach to eating behavior problems, it is important to determine the individual eating behavior disorder. Due to the increased risk of eating behavior disorder in those who work in long shifts such as 24 hours, it will be possible to organize work programs and increase the quality of service.
